# Socioeconomic factors influencing rural-urban ambulance response time disparities in Connecticut

**DOI:** 10.1007/s43999-024-00055-9

**Published:** 2024-12-04

**Authors:** Eashwar Krishna

**Affiliations:** University of Connecticut, Storrs, CT USA

**Keywords:** Rural health, Emergency medical services, Ambulances, Connecticut, Public Health

## Abstract

Across the U.S, it is a documented fact that rural areas have longer ambulance response times and tend to have lower median income. The objective of this study was to test if the rural-urban emergency medical service (EMS) response time disparity was related to wealth disparity in the state of Connecticut. All mean EMS response times were sourced from the 2016 Office of Emergency Medical Services Data Report. Rural definitions were sourced from the Connecticut Office of Rural Health. Median income data was drawn from the Connecticut Office of Policy and Management. A Mann-Whitney U test determined if the average rural EMS response time was greater than the non-rural EMS response time. Pearson coefficients quantified the relationship between median income and EMS response time. A t-test ascertained if the average median income differed between the two datasets. The mean EMS response time was 12.98 min (SD = 3.36) rural and 8.26 min (SD = 2.12) non-rural. Rural mean response time and median income were not significantly correlated (*r* = -.148, *p*=.247); non-rural mean response time and median income were also not significantly related. No significant disparity was detected (t=0.478, *p*=.633) between the mean rural household income ($98,258) and mean non-rural household income ($95,706). Significant disparities in EMS response times can exist between rural and non-rural towns separate from median income trends, as is the case in Connecticut. These findings may have limited generalizability because of Connecticut’s relatively high median income as compared to other states yet may be relevant to states with similar economic metrics.

**Supplementary Information:**

The online version contains supplementary material available at 10.1007/s43999-024-00055-9.

## Introduction

It has been demonstrated that shorter ambulance response times improve immediate and long-term outcomes during cardiac arrests [1, 2]; for example, O’Keefe et al. [3] found for every one minute reduced in response time, the chance of survival increased by 24%. While patient survival rates of an adverse cardiac event can be greatly improved through bystander education and intervention, the same cannot be said for other out-of-hospital medical emergencies. One such case is foreign body airway obstruction events: in only about 5–13% of cases are bystanders able to make a correct diagnosis and perform the appropriate maneuver; moreover, approximately 30–40% of all such events occur when the victim is alone [4]. On average, the victim has six minutes before they begin to suffer from hypoxia-related brain damage.

As such, it is a public health imperative to provide timely professional care to the acutely ill and injured. Mell et al. [5] report that across the USA, rural response times are almost double suburban and urban responses. A good amount of heterogeneity among the results of rural-urban response time disparity studies has also been documented [6], creating the need to study various regions of the United States in isolation from each other.

While the relationship between median income and response times [7], as well as rurality and response times [5], has been studied, the interrelation of these two factors has not yet been the subject of sufficient research. Connecticut, a northeastern state of the USA, is an excellent case study to understand the unique challenges that face rural EMS agencies and the role, if any, of median income. On a national level, urban counties tend to have higher median income than rural counties [8]; in contrast, Connecticut has the highest median income and lowest poverty rates for rural areas in the country [9], and possibly no significant rural-urban wealth disparity.

I hypothesized that a significant disparity between rural and non-rural response times will persist in Connecticut; however, I expected that this disparity would not be correlated with median income.

## Methods

All mean EMS response times were sourced from the 2016 Office of Emergency Medical Services Data Report, published by the Connecticut Department of Public Health. Towns, neighborhoods, or villages with fewer than thirty recorded EMS encounters, incomplete ZIP (Zone Improvement) code data, or incomplete time point data were excluded from the report [10]. It should be noted that many of these excluded areas were in fact neighborhoods under the jurisdiction of a recorded town. The only independent rural town without a mean response time reported due to insufficient reports was Lyme; Union, North Canaan, Sharon, and Warren were excluded from the report with no explicit explanation yet it may be assumed that they lacked complete time points or ZIP code data. Stafford, Griswold, and Killingly – classified for the purposes of this study as non-rural – were also excluded from the report due to incomplete data.

The Connecticut Office of Rural Health has published a list of towns considered rural according to the state definition, which was adopted for use in this study; other rural definitions such as RUCC and RUCA were not applicable to this study as national standards do not use towns as their fundamental units of study. Towns were chosen as the focus of this study because they are the smallest units of governance in Connecticut, and as such EMS policy and median income can vary widely between even neighboring towns in the same county.

All towns with a population of fewer than ten thousand residents as well as a population density of five hundred people or fewer per square mile were classified as rural [11], yielding 68 rural towns in total; 63 towns were used for data analysis due to the remaining five lacking data. Urban and suburban towns were grouped together since the nationwide mean times for both groups were highly similar; in total, 95 towns and cities were used for the non-rural comparison group. The dataset used in the analysis can be found in Appendix A and B.

Average household median income data was drawn from the Municipal Fiscal Indicators dataset released by the Connecticut Office of Policy and Management [12]. Median income and mean response time data were paired for each town, and the overall dataset was split into rural and non-rural sets. Only towns being used for the response time comparison were included in these datasets; the eight towns excluded for the aforementioned reasons were also excluded from the median income calculations. The median income sets can be found in Appendix C and D.

### Statistical analysis

The null hypothesis was that the distribution of mean response times (MRT) for rural and urban towns would be equal, whereas the alternative hypothesis was that the distributions would not be equal. A two-sample Wilcoxon-Mann-Whitney U test with (alpha) = 0.05 was performed to test if the rural distribution of response times was stochastically different than the urban distribution. The two datasets had different distribution shapes, and thus a non-parametric test was necessary. Measures of central tendency were calculated and the Tukey fence method was used to better describe the data and discover outliers, respectively.

Pearson coefficients of correlation were calculated for the relationship between average household median income and mean response time for both sets. Furthermore, a two-tailed t-test with (alpha) = 0.05 was performed to determine if median household income varied between the rural and non-rural datasets. The data were analyzed using IBM SPSS statistics (Version 29).

### Ethics approval

This study only analyzed publicly-available, de-identified data and as such did not meet the criteria for IRB review according to 45 CFR 46.102.

## Results

A total of 158 towns were included in the analysis. The mean response time for the rural dataset was 12.98 min (SD = 3.6), while the same for the non-rural data was 8.26 min (SD = 2.12); the distributions in the two groups differed significantly (z = 8.3464, *p* < .001). Median response times were 12.8 min and 7.7 min for rural and non-rural respectively. The observed standardized effect size was medium at 0.66, meaning that the magnitude of the difference between the two datasets was sizable. No outliers were observed in the rural data; Simsbury and Weston were non-rural dataset outliers with 15.8- and 14.6-minute mean response times, respectively, yet were still included in the analysis.

Neither rural mean response time and median income (*r* = − .148, *p* = .247) nor non-rural mean response time and median income (*r* = .137, *p* = .185) were found to be significantly correlated. There was no significant difference in average household median incomes between the two datasets (t = 0.478, *p* = .633).

## Discussion

While rural response times statewide are lower than the national rural average, a significant disparity still exists between non-rural and rural towns within Connecticut; this inequity has serious implications for patient outcomes. Though only a few urban/suburban towns have a mean response time below five minutes, generally considered the gold standard, the average non-rural MRT is not significantly far from it. In contrast, the average rural response time in Connecticut is almost thirteen minutes, well over double the time necessary to consistently save victims of traumatic injuries, choking, or other severe events.

In addition, the data makes it evident that median income has no statistically significant impact on response time within either dataset. Moreover, there is no significant wealth disparity as measured by household median income between rural and non-rural areas. This suggests that more focus should be directed towards other aspects of rural EMS, such as staffing shortages and town policies, rather than solely focusing on socioeconomic factors. It should be noted that this conclusion is specific to Connecticut; it may or may not be the case elsewhere that the most pertinent issue to response time disparity is a rural-urban wealth disparity. While lower-income towns may struggle with EMS procurement and operational costs, which can be unpredictable [13], more than higher-income towns, multiple papers have demonstrated that the impact of non-financial factors may be more influential in resolving the response time disparity. For example, Wei Lam et al. found that changes to dispatch policy and more effective allocation of existing ambulances would be more effective than merely adding more ambulances in reducing response times overall [14]. As for rural response times specifically, poor access to primary care for non-acute health management, provider burnout, and lack of opportunities for provider-initiated workplace improvements have been cited by Newton et al. as issues contributing to lengthy rural EMS response times [15]. The same study indicated that directing federal and state funding to community paramedicine initiatives may be effective at managing the underlying health disparities present in rural and remote communities; failure to provide consistent primary care to these populations tends to result in increased EMS utilization [16], which can put already-strained units out-of-service.

Other communities may find it beneficial to conduct small-scale, local investigations regarding EMS response in their jurisdiction. Much of the existing work on response time and rurality focuses on county [17] or ZIP codes [5] as geographic units of analysis; however, this is frequently not the level at which policy and quality improvement interventions are implemented. For example, in the New England region of the United States, which includes Connecticut, municipalities such as villages, towns, and cities regulate emergency services independently, with limited oversight from the state. The implication of this fact is that county-focused analysis may be erroneously grouping together a vastly heterogenous number of local EMS services, with varying degrees of funding, privatization, and quality improvement initiatives. Conversely, ZIP code-focused analysis may become too granular, as a single municipality frequently covers multiple ZIP codes. This study attempts to illustrate municipality-level differences, which emphasize the necessity of local policy innovations to combat long response times.

Such policy changes are already underway in Connecticut, and this study underscores their necessity and potential impact; since rural-urban response time disparities in the state do not appear to be correlated with median income, such disparities are more remediable by tailoring solutions to the unique challenges faced by rural communities. Rather than utilize limited funds to solely subsidize equipment purchases, it may be far more impactful to pursue EMS innovation and fundamental alterations to the existing pre-hospital care infrastructure. A prime example of such a project is the Mobile Integrated Health workgroup, chaired by the Connecticut Department of Public Health, which aims to create a community paramedicine initiative that will integrate with existing EMS systems. Notably, the group’s most recent report indicates a goal to “triage and connect non-urgent 9-1-1 callers with relevant caregivers and assistance instead of dispatching an ambulance crew” [18]. The results of this study in combination with existing research indicate that it will be efficacious for additional attention to be given to community-level interventions in rural areas such as health literacy programs, increased incentives for primary care providers, and increased expansion of mobile integrated health solutions in Connecticut.

### Limitations

In order to accurately capture the effect of median income and town policy, analysis was conducted by town lines; however, the source data for mean response time did not classify towns by strict jurisdictional borders, and as such included neighborhoods or villages of towns as separate entries. In these situations, the town center data point was used for mean response time; as such, the fidelity of the data is a limitation of this research. This study also limited its analysis of socioeconomic factors to median household income, and other such factors may affect response times.

The data utilized in this study, collected in 2016, may present certain implications for the results. The age of the data is a critical factor to consider, as it may not fully capture recent changes in EMS infrastructure, technological advancements, or policy shifts that have occurred since then. For instance, while rural towns have predominantly relied on volunteer services for coverage, a declining number of volunteers has led some of these towns to contract private agencies. Subsequently, these municipalities reported decreased response times [19]. Such trends, if significant across most towns included in the rural dataset, may be hypothesized to decrease the disparity in response time observed in this study.

Despite these shifts, a more recent report from the Department of Public Health (DPH) indicates that the overall environment of EMS as it relates to rural-urban response time disparities has remained roughly the same. At the time of the analysis, the non-rural mean response time (MRT) was 8.26 min; this report was published five years later and reports a suburban MRT of 8.12 min and an urban MRT of 7.39 min [20]. Rural towns were noted to have a mean response time of 10.07 min in the more recent report, compared to 12.8 min in this study. This disparity can be explained by data reporting standards changing; namely, at the time of the data analysis in this study, median response times were not stratified by rural status by the DPH. In the more recent report, several towns that were previously under the non-rural dataset have been included in the rural MRT average, which understandably lowers this number. While this phenomenon is partially explained by population trends and possibly differences in methodology, a repetition of the analysis in this study with newer data would be warranted if town-specific conclusions need to be drawn. However, the general trend illustrated by this study and the interpretation thereof remains relevant to both the state of Connecticut as well as other communities who might benefit from investigating novel solutions to the indisputable disparity in rural pre-hospital care.

## Conclusion

 There exists a significant rural-urban disparity in ambulance response times in Connecticut which is decoupled from median income, a concerning trend that demands amelioration in the near future to ensure equitable access to timely healthcare. The most effective strategy to do so will be one that focused on policy and innovation in both emergency medical services as well as care focusing on chronic and preventable conditions.

## Electronic supplementary material

Below is the link to the electronic supplementary material.

Supplementary Material 1

## Data Availability

Data was publically available through the Connecticut Department of Public Health.
