# Supplementary material for: Socioeconomic factors influencing rural-urban ambulance response time disparities in Connecticut
Source: Res Health Serv Reg. 2024 Dec 4;3:19. doi: 10.1007/s43999-024-00055-9 (PMC11615172; doi:10.1007/s43999-024-00055-9)
Supplement: Supplementary file 1 — Supplementary Material 1 [file 43999_2024_55_MOESM1_ESM.docx]

Socioeconomic Factors Influencing Rural-Urban Ambulance Response Time Disparities in Connecticut

Research in Health Services and Regions

Eashwar Krishna, University of Connecticut

eashwar.krishna@uconn.edu

**Appendix A: Rural Towns and Mean Response Time**

Rural towns and their reported mean response time (MRT) according to Connecticut Department of Public Health data. Names in parentheses represent the name used by the report, which may differ from the name of the jurisdictional town.

| **Town** | **MRT (min)** | **Town** | **MRT (min)** |
| --- | --- | --- | --- |
| Andover | 11.7 | Deep River | 14.7 |
| Bolton | 9.5 | Durham | 12.2 |
| Columbia | 9.3 | East Haddam | 20.5 |
| Hebron | 12.4 | Haddam | 14.5 |
| Willington | 10.1 | Killingworth | 17.5 |
| Bozrah | 12 | Middlefield | 9.3 |
| Franklin (North Franklin) | 12.9 | Portland | 8.8 |
| Lebanon | 15 | Westbrook | 21.3 |
| Lisbon | 8.2 | Burlington | 9.8 |
| North Stonington | 12.9 | East Granby | 8.5 |
| Old Lyme | 14.3 | Hartland (East Hartland)  (West Hartland < 30 reports) | 13.8 |
| Preston | 10.6 | Marlborough | 22 |
| Salem | 11.9 | Sherman | 16.5 |
| Sprague (Baltic) | 11.9 | Redding | 10.4 |
| Voluntown | 18 | Easton | 8.8 |
| Bethany | 12.3 | Scotland | 11.4 |
| Middlebury | 10.1 | Canterbury | 17.2 |
| Woodbridge | 7.6 | Sterling | 16.1 |
| Chester | 15.8 | Chaplin | 10 |

| Brooklyn | 10.7 | Morris | 12.8 |
| --- | --- | --- | --- |
| Hampton | 13.7 | Bethlehem | 16.6 |
| Pomfret | 15.8 | Woodbury | 16.9 |
| Ashford | 12.7 | Roxbury | 13.9 |
| Eastford | 15.4 | Bridgewater | 12.8 |
| Putnam | 6.8 | Washington | 17 |
| Thompson | 11.2 | Colebrook | 12.8 |
| Woodstock | 11.3 | Barkhamsted | 13.9 |
| Salisbury | 14.9 | Cornwall | 15.3 |
| Canaan | 8.6 | Goshen | 14.1 |
| Norfolk | 13 | New Hartford | 12.7 |
| Kent | 16.6 | Harwinton | 9.1 |
| Litchfield | 9 |  |  |

**Appendix B: Non-Rural Towns and Mean Response Time**

Urban/suburban towns and their reported mean response time (MRT) according to Connecticut Department of Public Health data. Names in parentheses represent the name used by the report, which may differ from the name of the jurisdictional town.

| **Town** | **MRT (min)** | **Town** | **MRT (min)** |
| --- | --- | --- | --- |
| Bridgeport | 7.1 | Southington | 7 |
| Stamford | 7.4 | Enfield | 7.2 |
| New Haven | 7.7 | Shelton | 9.6 |
| Hartford | 6.6 | Norwich | 6.4 |
| Waterbury | 7.2 | Groton | 7.8 |
| Norwalk | 8.3 | Trumbull | 8.5 |
| Danbury | 6.8 | Torrington | 6.1 |
| New Britain | 6.7 | Glastonbury | 6.1 |
| West Hartford | 6.3 | Naugatuck | 7.7 |
| Greenwich | 5.5 | Newington | 6.5 |
| Fairfield | 6.5 | Vernon (Vernon Rockville) | 7.2 |
| Hamden | 10.8 | Windsor | 8.2 |
| Meriden | 5.5 | Cheshire | 7 |
| Bristol | 12.3 | Branford | 7 |
| Manchester | 6.2 | New Milford | 10.2 |
| West Haven | 9.9 | East Haven | 8.6 |
| Stratford | 7.5 | New London | 4.8 |
| Milford | 7.7 | Wethersfield | 5.7 |
| East Hartford | 6.3 | Newtown | 10.1 |
| Middletown | 6.6 | Westport | 7.5 |
| Wallingford | 7.8 | South Windsor | 6.8 |

| Farmington | 7.2 | Montville | 6.1 |
| --- | --- | --- | --- |
| Mansfield | 8.5 | Stonington | 8.8 |
| Ridgefield | 7 | Madison | 6.3 |
| Simsbury | 15.8 | Brookfield | 6.6 |
| Windham | 8.3 | Plainville | 7.7 |
| North Haven | 8.2 | Seymour | 8.1 |
| Watertown | 10.6 | Ellington | 8.9 |
| Guilford | 8.5 | Wolcott | 6.6 |
| Bloomfield | 11.5 | Suffield | 9.6 |
| Darien | 6.6 | Colchester | 10.1 |
| Rocky Hill | 6.6 | Ledyard | 7.3 |
| New Canaan | 7.2 | Plainfield | 10.7 |
| Bethel | 6.2 | Tolland | 10.9 |
| Berlin | 7 | Orange | 8.2 |
| Southbury | 9.5 | Cromwell | 8.2 |
| Waterford | 7.2 | New Fairfield | 10.7 |
| Avon | 8 | North Branford | 12.4 |
| Ansonia | 6 | Clinton | 12.3 |
| Monroe | 9.6 | East Hampton | 12.1 |
| East Lyme | 6.3 | Oxford | 11.6 |
| Wilton | 8.3 | Windsor Locks | 5.2 |

| Derby | 7.1 | East Windsor | 6.1 |
| --- | --- | --- | --- |
| Coventry | 10.4 | Granby | 8 |
| Plymouth | 9.5 | Old Saybrook | 11.1 |
| Weston | 14.6 | Canton | 11 |
| Somers | 8.6 | Prospect | 11.8 |
| Winchester | 11 |  |  |
|  |  |  |  |
|  |  |  |  |

**Appendix C: Rural Towns and Median Income**

Rural towns and their average household median income according to the CT Office of Policy and Management. Names in parentheses represent the name used by the report, which may differ from the name of the jurisdictional town.

| **Town** | **Median Income (USD)** | **Town** | **Median Income (USD)** |
| --- | --- | --- | --- |
| Andover | 99449 | Deep River | 80495 |
| Bolton | 112622 | Durham | 130635 |
| Columbia | 104911 | East Haddam | 95685 |
| Hebron | 126045 | Haddam | 107073 |
| Willington | 73264 | Killingworth | 104462 |
| Bozrah | 91838 | Middlefield | 79042 |
| Franklin | 91875 | Portland | 97754 |
| Lebanon | 97422 | Westbrook | 73988 |
| Lisbon | 86641 | Burlington | 129783 |
| North Stonington | 61963 | East Granby | 107566 |
| Old Lyme | 105417 | Hartland | 97850 |
| Preston | 87885 | Marlborough | 113547 |
| Salem | 104725 | Sherman | 120682 |
| Sprague | 72989 | Redding | 135928 |
| Voluntown | 84583 | Easton | 166875 |
| Bethany | 151034 | Scotland | 88077 |
| Middlebury | 120480 | Canterbury | 86178 |
| Woodbridge | 169155 | Sterling | 84643 |
| Chester | 87717 | Chaplin | 83393 |

| Brooklyn | 75993 | Morris | 82240 |
| --- | --- | --- | --- |
| Hampton | 76364 | Bethlehem | 92237 |
| Pomfret | 91788 | Woodbury | 100850 |
| Ashford | 84909 | Roxbury | 109063 |
| Eastford | 96625 | Bridgewater | 130186 |
| Putnam | 64320 | Washington | 102114 |
| Thompson | 75168 | Colebrook | 96250 |
| Woodstock | 92165 | Barkhamsted | 103500 |
| Salisbury | 72632 | Cornwall | 90197 |
| Canaan | 68750 | Goshen | 117438 |
| Norfolk | 75179 | New Hartford | 103400 |
| Kent | 77344 | Harwinton | 110652 |
| Litchfield | 87241 |  |  |

**Appendix D: Non-Rural Towns and Median Income**

Urban/suburban towns and their average household median income according to the CT Office of Policy and Management. Names in parentheses represent the name used by the report, which may differ from the name of the jurisdictional town.

| **Town** | **Median Income (USD)** | **Town** | **Median Income (USD)** |
| --- | --- | --- | --- |
| Bridgeport | 47484 | Southington | 101098 |
| Stamford | 96885 | Enfield | 83232 |
| New Haven | 44507 | Shelton | 98873 |
| Hartford | 36154 | Norwich | 57565 |
| Waterbury | 46329 | Groton | 68849 |
| Norwalk | 75179 | Trumbull | 129239 |
| Danbury | 73204 | Torrington | 60662 |
| New Britain | 47393 | Glastonbury | 120605 |
| West Hartford | 105230 | Naugatuck | 77967 |
| Greenwich | 167537 | Newington | 82721 |
| Fairfield | 140308 | Vernon (Vernon Rockville) | 67492 |
| Hamden | 78602 | Windsor | 92079 |
| Meriden | 58472 | Cheshire | 122477 |
| Bristol | 68485 | Branford | 80471 |
| Manchester | 74270 | New Milford | 93815 |
| West Haven | 64255 | East Haven | 67773 |
| Stratford | 82286 | New London | 47424 |
| Milford | 95627 | Wethersfield | 90881 |
| East Hartford | 59954 | Newtown | 125028 |
| Middletown | 62022 | Westport | 222375 |
| Wallingford | 83054 | South Windsor | 119972 |

| Farmington | 97262 | Montville | 80765 |
| --- | --- | --- | --- |
| Mansfield | 50492 | Stonington | 93349 |
| Ridgefield | 152630 | Madison | 119777 |
| Simsbury | 128829 | Brookfield | 117292 |
| Windham | 42909 | Plainville | 70012 |
| North Haven | 104812 | Seymour | 80396 |
| Watertown | 71964 | Ellington | 89645 |
| Guilford | 108243 | Wolcott | 96014 |
| Bloomfield | 79134 | Suffield | 110938 |
| Darien | 243750 | Colchester | 107914 |
| Rocky Hill | 85123 | Ledyard | 92439 |
| New Canaan | 200203 | Plainfield | 66689 |
| Bethel | 94973 | Tolland | 118367 |
| Berlin | 102075 | Orange | 129489 |
| Southbury | 102128 | Cromwell | 89562 |
| Waterford | 90670 | New Fairfield | 114583 |
| Avon | 123077 | North Branford | 96019 |
| Ansonia | 53709 | Clinton | 90332 |
| Monroe | 121847 | East Hampton | 95663 |
| East Lyme | 96023 | Oxford | 106089 |
| Wilton | 204473 | Windsor Locks | 72090 |

| Derby | 58534 | East Windsor | 78667 |
| --- | --- | --- | --- |
| Coventry | 93619 | Granby | 111347 |
| Plymouth | 79029 | Old Saybrook | 86802 |
| Weston | 207863 | Canton | 89863 |
| Somers | 106305 | Prospect | 106667 |
| Winchester | 63882 |  |  |
|  |  |  |  |
|  |  |  |  |
